# Prognosis and risk factors in older patients with lung cancer and pulmonary embolism: a propensity score matching analysis

**DOI:** 10.1038/s41598-020-58345-4

**Published:** 2020-01-27

**Authors:** Liu Junjun, Wang Pei, Yan Ying, Song Kui

**Affiliations:** Department of Oncology, Beijing Hospital, National Center of Gerontology, Institute of Geriatric Medicine, Chinese Academy of Medical Science, Beijing, 100730 China

**Keywords:** Cardiovascular diseases, Lung cancer

## Abstract

Older patients, especially those with malignancy, may have an increased risk of pulmonary embolism (PE). However, few studies have evaluated the clinical characteristics and prognosis of older patients. We evaluated the clinical characteristics, prognosis, and risk factors in older patients with lung cancer complicated with PE. This was a single-center, prospective cohort study. Older patients (≥65 years) with lung cancer admitted in Beijing Hospital from January 2006 to December 2016 were enrolled. The patients were divided into two groups according to the presence of PE using propensity score matching (PSM). After PSM, one hundred and six patients (53 per group) with an average age of (77.3 ± 10.9) years were enrolled. Adenocarcinoma was the most common histology in patients with PE (52.8%, n = 28), and most lung cancer patients were in stages III and IV (59.4%, n = 63). Patients with PE were stratified to low risk (52.8%, n = 28), intermediate-low risk (24.5%, n = 13), intermediate-high risk (15.1%, n = 8), high-risk (7.5%, n = 4) subgroups. Most PE patients presented with dyspnea (75.5%), and the majority of patients (86.8%, n = 46) developed PE within 3 months after the diagnosis of cancer. The median follow-up time was 23.7 months (12.0–62.0 months), and 7 patients (6.6%) were lost to follow-up. During the follow-up period, 92 patients (86.8%) died, including 8 cases (8.7%) of PE-related death, 73 (79.3%) of tumor death, and 11 (11.9%) of unknown cause. There were significant differences in all-cause mortality (94.3% *vs*. 83.0%) and PE-related mortality (15.1% *vs*. 0) between the PE and control groups, but the rate of tumor-related mortality (75.5% *vs*. 66.0%) was comparable between the groups. Among the 92 patients who died, the mortality rates at 3, 6, 12, and > 12 months after tumor diagnosis were 33.0% (33/106), 57.5% (61/106), 78.3% (83/106), and 89.6% (95/106), respectively. Kaplan–Meier survival analysis showed that the median overall survival time was significantly different between the PE and the control groups (4.3 *vs*. 9.2 months, *P* = 0.0015). Multivariate stepwise logistic regression analysis showed that age ≥ 77 years (*OR* = 2.58, 95%*CI*: 1.66–4.01), clinical stage III–IV (*OR* = 2.21, 95%*CI*: 1.03–4.74), adenocarcinoma (*OR* = 3.24, 95%*CI*: 1.75–6.00), high D-dimer (≥600 mg/L) (*OR* = 2.73, 95%*CI*: 1.25–5.96), and low partial pressure of oxygen (PaO_2_; <75 mmHg) (*OR* = 2.85, 95%*CI*: 1.74–4.67) were independent risk factors for PE in older patients with lung cancer. Older patients with lung cancer and PE often have poor prognosis. Advanced age, clinical stage III–IV, adenocarcinoma, high D-dimer level, and low PaO_2_ are independent risk factors for PE.

## Introduction

With age, older individuals are at high risk of thromboembolic events. Cancer is an established cause of venous thrombosis^1^; once a thrombus forms, venous thromboembolism (VTE) and even pulmonary embolism (PE) can result, leading to serious adverse events such as heart failure, respiratory failure, and sudden death. The prevalence of malignancy in patients with PE ranges between 4% and 20%, and almost 50% of patients with tumors have thromboses on autopsy^2,3^. The overall risk of PE is increased twenty-fold in patients with tumors compared with the general population^4^. In a retrospective study in 435 patients with cancer, the incidence of PE was 3.3% and the mortality rate was as high as 30%^5^. Thrombotic events are the second leading cause of death in patients with tumors^6^. In our hospital, 1.51% (280/18531) of patients with cancer present with (VTE), most occur within the first 6 months after cancer diagnosis and nearly half of deaths occur within the first 3 months of VTE diagnosis^7^. Lung cancer is the sixth most common malignancy coexisting in patients with PE^8,9^. Therefore, early diagnosis and timely treatment of thrombosis is warranted in this patient population.

However, clinical manifestations of older patients with lung cancer and PE are atypical. The National Central Cancer Registry of China (NCCR) recently reported that lung cancer was the most common cancer and the leading cause of tumor mortality in men aged 60 years and above^10^. Most new cancer cases and tumor mortality in men occur in the age range of 60–74 years^11^. The typical presentation forms in older patients with lung cancer complicated with PE include dyspnea, chest pain, and/or hemoptysis. In our previous study, we found that PE in patients with cancer frequently causes dyspnea (51.5%)^7^. Compared with the younger patients, the main symptoms presented in elderly patients are dyspnea (73.4% vs 63,7%), chest pain (26.6% vs 39.5%) and cough/hemoptysis (31.3% vs 18.9%)^12^. Therefore, missed diagnosis and misdiagnosis for PE remain relatively common in older patients. Older patients have more comorbidities, a weak organ compensatory capacity, and poor prognosis. In-hospital (13% vs 2%) and long-term (36% vs 12%) mortality rates are significantly higher in elderly patients compared with the younger population with PE^13^. Furthermore, lung cancer complicated with PE may significantly increase treatment difficulties and health-care expenditure, reduce quality of life, and shorten survival time^14^. Recently, Lange *et al*. studied 991 older patients (≥65 years) with acute VTE in a Swiss prospective cohort study, and found that multimorbid older patients with VTE have not only a lower anticoagulation quality but only more complications^15^, and only 12% of patients with a malignancy complicated with VTE survive beyond 1 year^16^. In our hospital, the median survival time of patients with cancer and complicated with isolated PE was 16.0 ± 10.5 months^7^. However, owing to limited data, few studies have evaluated the clinical characteristics and prognosis in older patients with comorbidities of PE and cancer. Therefore, we aimed to evaluate the clinical characteristics, prognosis, and risk factors in older patients with lung cancer complicated with PE.

## Methods

### Ethical approval

This study was conducted in accordance with the Declaration of Helsinki and was approved by the Institutional Review Board of Beijing Hospital. Written informed consent was obtained from all patients.

### Study design and population

This was a single-center, prospective cohort study. Older patients aged 65 and more years old with lung cancer and admitted in Beijing Hospital were enrolled. Patients who had lung cancer with PE were regarded as the PE group and patients without PE were regarded as the control group using propensity score matching.

### Inclusion criteria

(1) Patients aged 65 and more years old; (2) lung cancer was diagnosed by histological or cytological examination; (3) PE and risk classification was diagnosed in line with the PE diagnosis and treatment guidelines of the Chinese Thoracic Society.

### Exclusion criteria

(1) Primary tumor other than lung cancer; (2) diagnosis of PE prior to admission; (3) presence of severe liver failure, renal failure, or other organ dysfunction; (4) incomplete data.

### Procedures

Patients’ medical information were collected from the hospital information system or medical record system. A self-designed data collection form was used to record demographic data, medical history, laboratory examination, and CT pulmonary angiography features. Tumor-related data included tumor histology, clinical stage, and treatment modality. PE-related conditions included time between PE diagnosis and lung cancer diagnosis, symptoms, risk stratification, and treatments^7^. All clinical characteristics of DVT were collected within 1 month of diagnosis.

### Follow-up

All patients were followed up, mainly by telephone communication and outpatient visit. Follow-up data were recorded until December 2017 or death. Date of death was confirmed from registration data of the Department of Civil Affairs of China. Survival time was defined as the time from the diagnosis until death or truncation, which included patients who were lost to follow-up or still alive at the time of study termination^7^. The primary endpoint was the overall survival rate. The secondary endpoint was the tumor-related mortality rate and PE-related mortality rate.

### Statistical analyses

One-to-one matching was undertaken to overcome potential selection bias by the propensity score matching (PSM) method between the two groups. Using the multiple logistic regression analysis, a propensity score was estimated for all patients. Variables used in the model included age, sex, BMI, smoking, medical history, D-dimer, WBC, hemoglobin, platelet, PaO_2_, hemodynamic parameters, clinical TNM stage, histology and treatment. We performed caliper matching on the propensity score (nearest available matching). Pairs on the propensity score logit were matched within a range of 0.2 SD. Matching was performed by the minimal adjacent method of 1:1 pairing^17^.

All patients were included in the analysis according to the intention-to-treat principle. Data were expressed as the mean and standard deviation for normally distributed continuous variables, and as absolute number and percentage for categorical variables. Statistical differences were analyzed using the t-test for continuous data and the χ2 test for categorical data. Kaplan–Meier survival analysis was used to evaluate the median survival time, and the log-rank test was used to determine the statistical difference between groups. After univariate analysis, all variables with *P* values less than 0.15 were considered in subsequent multivariate analyses. The odds ratio (OR value) and corresponding 95% confidence interval (CI) was calculated using stepwise multivariate logistic regression analysis to evaluate the independent risk factors for PE. A *P* value < 0.05 was considered statistically significant. All statistical tests were two-sided. Data processing was performed using TATA software (version 12.0; StataCorp, College Station, TX, USA).

## Results

### Baseline characteristics

**Figure 1:**
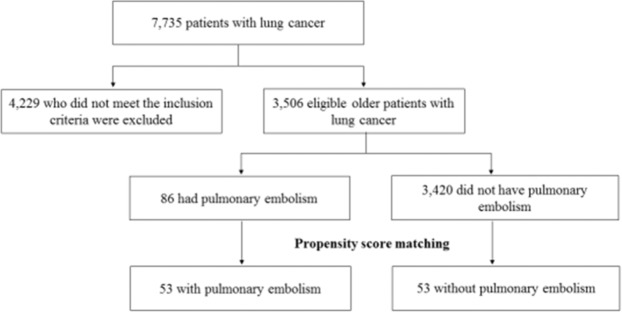
Study flow diagram.

From January 2006 to December 2016, 7,735 patients with lung cancer were admitted to our hospital. Patients who did not meet the inclusion criteria and those with incomplete data (n = 4,229) were excluded from the analysis. There were 86 older patients with lung cancer and PE and 3,420 older patients without PE. After propensity score matching, 53 patients were categorized as the PE group and 53 patients as the control group (Fig. 1).

Among the 106 older patients with lung cancer, 57 were male, and the average age was (77.3 ± 10.9) years (65–90 years). Baseline characteristics such as age, sex, body mass index, smoking and risk factors, platelets and hemoglobin, clinical stage, risk stratification, and treatment were similar between the PE group and the control group (*P* > 0.05).

Among patients with cancer and PE, adenocarcinoma was the most common histology (52.8%, n = 28), and most lung cancer patients were in stages III and IV (59.4%, n = 43). All 53 patients with PE were stratified to low risk (52.8%, n = 28), intermediate-low risk (24.5%, n = 13), intermediate-high risk (15.1%, n = 8), or high-risk (7.5%, n = 4) subgroups. In addition, most patients with PE (75.5%, n = 40) presented with dyspnea, 5 cases (9.4%) presented with chest pain, 2 cases (3.8%) presented with syncope, and 6 cases (11.3%) were asymptomatic. In the PE group, 31 patients had DVT, mostly in the lower extremities (n = 24). For the majority of patients with PE (86.8%, n = 46), PE appeared within 3 months after the diagnosis of cancer. Thirty-seven (69.8%) patients received low-molecular-weight heparin and/or warfarin anticoagulant therapy, 6 patients received intravenous recombinant tissue plasminogen activator (rt-PA) thrombolysis, and 9 patients did not receive anticoagulant or thrombolytic therapy because of contraindications (Table 1).

**Table 1:** Baseline characteristics of older patients with or without PE.

Data	Overall cohort				Propensity score matched cohort			
	PE group (n = 86)	Control group (n = 3,420)	*t/χ*^2^ value	*P* value	PE group (n = 53)	Control group (n = 53)	*t/χ*^2^ value	*P* value
**Basic characteristics**								
Age(years)	79.3 ± 12.7	75.2 ± 9.1	4.080	<0.001	77.4 ± 10.8	77.2 ± 6.4	0.647	0.519
Male	58(67.4%)	2094(61.2%)	1.397	0.237	29(54.7%)	28(52.8%)	0.038	0.846
BMI (kg/m^2^)	22.7 ± 4.9	23.5 ± 4.2	1.737	0.083	22.1 ± 3.5	22.5 ± 2.7	−0.659	0.512
Smoking	52(60.4%)	1941(56.8%)	0.474	0.491	37(69.8%)	33(62.3%)		
**Medical history**								
Hypertension	31(36.0%)	1345(39.3%)	0.383	0.536	17(32.1%)	19(35.8%)	0.168	0.682
Diabetes	35(40.7%)	989(28.9%)	5.659	0.017	15(28.3%)	17(32.1%)	0.179	0.672
COPD	42(48.8%)	1294(37.8%)	4.192	0.041	33(62.3%)	31(58.5%)	0.158	0.691
Atrial fibrillation	22(25.6)	603(17.6)	3.619	0.057	18(34.0%)	10(18.9%)	3.106	0.078
Varicose veins	26(30.2%)	665(19.4%)	5.541	0.019	19(35.8%)	13(24.5%)	1.389	0.239
Deep vein catheterization	36(41.8%)	1073(31.4%)	4.072	0.044	18(34.0%)	14(26.4%)	0.718	0.397
DVT	44(51.2%)	295(8.6%)	99.394	<0.001	31(58.5%)	29(54.7%)	0.154	0.695
**Lab. test**								
D-Dimer (ug/L)	601.3 ± 282.2	399.5 ± 375.4	4.949	<0.001	532.3 ± 162.4	489.9 ± 185.7	1.251	0.214
WBC count (×10^9^/L)	8.92 ± 5.64	8.21 ± 5.97	1.088	0.277	8.6 ± 3.7	8.5 ± 4.1	0.132	0.895
Hemoglobin (g/L)	110.6 ± 49.3	131.2 ± 75.7	2.510	0.012	111.6 ± 26.8	127.7 ± 32.5	2.782	0.401
Platelet count (×10^9^/L)	182.3 ± 137.4	237.4 ± 199.8	2.544	0.011	226.6 ± 86.2	234.5 ± 99.1	0.438	0.662
PaO_2_ (mmHg)	68.2 ± 33.8	76.7 ± 32.7	2.379	0.017	72.2 ± 21.3	79.7 ± 12.4	1.233	0.220
**Hemodynamic parameters**								
SBP (mmHg)	107.8 ± 44.5	125.4 ± 42.7	3.771	0.0002	109.7 ± 12.3	113.6 ± 15.3	1.446	0.151
DBP (mmHg)	62.4 ± 26.3	70.6 ± 17.5	4.227	<0.001	68.5 ± 10.5	71.4 ± 8.5	1.563	0.121
Heart rate	113.7 ± 47.5	93.2 ± 43.7	4.287	<0.001	101.6 ± 13.3	98.2 ± 9.6	1.509	0.134
Clinical TNM stage			12.013	0.002			1.851	0.396
I-II	11(12.8%)	427(3.7%)			16(30.2%)	10(18.9%)		
III	41(47.7%)	1870(54.7%)			23(43.4%)	27(50.9%)		
IV	34(39.5%)	1123(41.6%)			14(26.4%)	16(30.2%)		
Histology			6.324	0.042			1.325	0.516
Adenocarcinoma	47(54.6%)	1974(58.0%)			28(52.8%)	23(43.4%)		
Squamous cell carcinoma	22(25.6%)	1081(31.6%)			17(32.1%)	18(34.0%)		
Large cell carcinoma	17(19.8%)	365(10.4%)			8(15.1%)	12(22.6)		
**Treatment (%)**								
Surgery	33(38.3%)	973(26.7%)	3.820	0.051	20(37.7%)	19(35.9%)	0.041	0.840
Chemotherapy	83(96.5%)	3176(92.9%)	2.057	0.151	51(96.2%)	50(94.3%)	0.211	0.646
Targeted therapy	24(27.9%)	745(21.7%)	1.735	0.188	13(24.5%)	15(28.3%)	0.194	0.659
Radiation	35(37.2%)	1026(30.0%)	1.993	0.158	16(30.2%)	18(34.0%)	0.173	0.677
Anti-coagulation	61(70.9%)	155(4.5)	256.699	<0.001	37(69.8%)	28(52.8%)	3.767	0.052
Thrombolysis	12(13.9%)	29(0.8)	42.381	<0.001	7(13.2%)	2(3.8%)	3.200	0.074

PE, pulmonary embolism; BMI, body mass index; COPD, chronic obstructive pulmonary disease; WBC, white blood cells; PaO2, partial pressure of oxygen; SBP, systolic blood pressure; DBP, diastolic blood pressure.

### Prognosis

**Figure 2:**
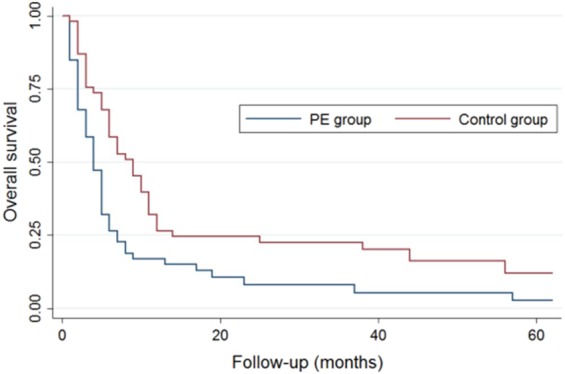
Kaplan–Meier survival analysis of median overall survival time.

**Figure 3:**
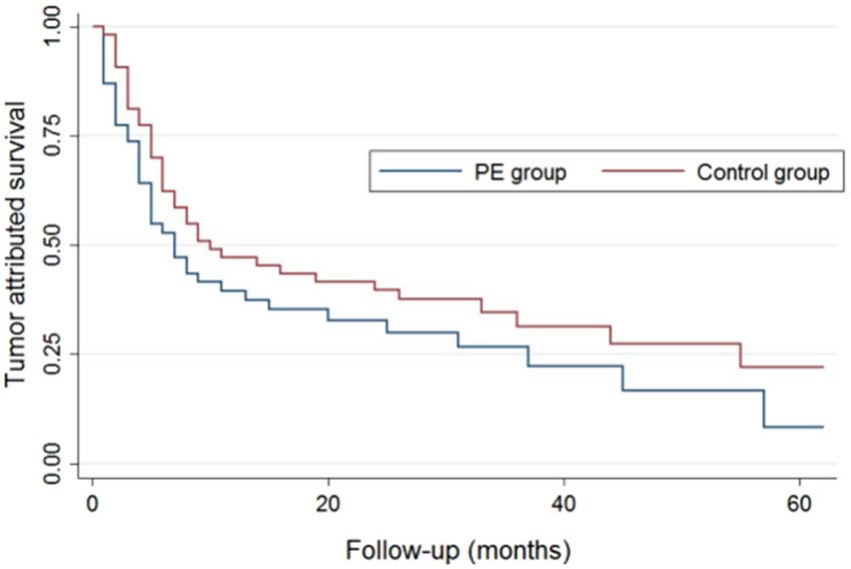
Kaplan–Meier survival analysis of tumor-related survival time.

The median follow-up time was 23.7 months (12.0–62.0 months) and 7 patients (6.6%) were lost to follow-up, including 5 in the PE group and 2 in the control group. During the follow-up period, 92 patients (86.8%) died, including 8 cases (8.7%) of PE-related death, 73 cases (79.3%) of tumor-related death, and 11 cases (11.9%) of unknown cause. There were significant differences in all-cause mortality and PE-related mortality between the PE and the control groups, but the rate of tumor-related mortality was comparable between the two groups. Among the 92 patients who died, the mortality rates at 3, 6, 12, and >12 months after tumor diagnosis were 46(43.4%), 18 (16.9%), 13 (12.2%), and 15 (14.2%), respectively. Kaplan–Meier survival analysis showed that the median overall survival time was significantly shorter in the PE group compared with the control group (4.3 *vs*. 9.2 months, *P* = 0.0015). However, the median tumor-related survival time was similar between the two groups (7.1 months *vs*. 9.7 months, *P* = 0.165) (Figs. 2 and 3).

### Risk factor related to PE of all patients

Multivariate stepwise logistic regression analysis was used to identify independent risk factors for PE. Age ≥ 77 years (*OR* = 2.58, 95%*CI*: 1.66–4.01), clinical stage III–IV (*OR* = 2.21, 95%*CI*: 1.03–4.74), adenocarcinoma (*OR* = 3.24, 95%*CI*: 1.75–6.00), high D-dimer level (≥600 mg/L) (*OR* = 2.73, 95%*CI*: 1.25–5.96), and low PaO_2_ (<75 mmHg) (*OR* = 2.85, 95%*CI*: 1.74–4.67) were identified as independent risk factors for PE (Table 2).

**Table 2:** Risk factors for PE by multivariate logistic analysis.

Risk factor	β	SE	Wald χ2	*OR*	95%CI	*P* value
Age ≥77 years	0.948	0.225	17.752	2.58	1.66–4.01	<0.001
Stage III-IV	0.793	0.390	4.134	2.21	1.03–4.74	0.042
Adenocarcinoma	1.176	0.314	14.027	3.24	1.75–6.00	0.002
D-Dimer ≥600ug/L	1.004	0.399	8.771	2.73	1.25–5.96	0.011
PaO_2_ < 75 mmHg	1.047	0.252	17.262	2.85	1.74–4.67	<0.001

PE, pulmonary embolism; PaO_2_, partial pressure of oxygen.

## Discussion

Older patients with lung cancer are susceptible to multiple risk factors for PE, including (1) patient-related factors such as advanced age, immobilization, hyperglycemia, hyperlipidemia, and smoking^18^; (2) malignant cell-related factors^19^, including the production of procoagulant, fibrinolytic, and proaggregating conditions; release of proinflammatory and proangiogenic cytokines; and interaction with vascular and blood cells; (3) treatment-related factors such as radiotherapy^20^, chemotherapy^21^, surgery^22^, and central venous catheterization^23^; and (4) complications^24^ such as infection, heart failure, renal disease, and pulmonary disease. Therefore, to the risk of PE should be considered in older patients with a malignancy.

Most older patients with lung cancer complicated with PE have no specific presentation. In a previous study enrolled 24 patients with lung cancer and PE, Chuang *et al*. showed that the most common symptoms were dyspnea (95.8%), hemoptysis (20.8%), cough (20.8%), chest pain (16.7%), body weight loss (8.3%), lower leg edema (8.3%), and shock (8.3%), which are not specific to lung cancer^25^. In another study enrolled 28 cases with lung cancer having PE and 56 cases with lung cancer alone, Li *et al*. found that 7.1% of patients with lung cancer complicated with PE were asymptomatic, and the incidence of cough (75.0% *vs*. 66.1%), stethalgia (25.0% *vs*. 7.1%), hemoptysis (14.3% *vs*. 19.6%), syncope (3.6% *vs*. 2.0%), palpitation (28.6% *vs*. 17.9%), and triad syndrome (3.6% *vs*. 0%) were similar between patients having lung cancer complicated with or without PE^26^. García Gómez LC conducted a hospital-based, case-control study included 413 patients with PE, 124 of whom were 80 years or older. Compared with the younger patients (<80 years), the main symptoms presented in elderly patients are dyspnea (73.4% vs 63,7%), chest pain (26.6% vs 39.5%) and cough/hemoptysis (31.3% vs 18.9%) [11]. Therefore, older patients with PE are less likely to present with chest pain and more likely to be hypoxic or have syncope^27,28^. In our study, 75.5% of older patients with PE presented with dyspnea, 9.4% with chest pain, and 3.8% with syncope, while 11.3% were asymptomatic. Moreover, typical symptoms of PE may disappear over time, giving rise to missed and delayed diagnosis^29^. Therefore, older patients with lung cancer complicated with PE are more likely to have common symptoms of lung cancer.

In the current study, patients with PE had a poorer prognosis. PE is a potentially fatal complication of venous thrombosis in patients with cancer. In 2010, the overall age-adjusted PE mortality rate was 21.0 per 100000. From 2000 to 2010, PE mortality declined in men and women over 55 years^30^. Meanwhile, in normotensive patients with PE with no evidence of right ventricular dysfunction, short-term mortality was 2%; while mortality rate rose to 30% in patients with shock and up to 65% in those with cardiac arrest^31^. In our study, 8 patients died of PE, including 5 patients in the intermediate-risk, 2 patients in the high-risk subgroups who died at 3 months of follow-up and 1 patient in the intermediate-risk subgroup who died at 6 months of follow-up. In previous studies, PE-related mortality in patients with lung cancer varied between 0.6% and 10%^26,32^, which was comparable with the 8.7% of PE-related mortality PE in our study. In addition, the effect of survival has been shown to be more prominent in patients who develop PE early in the course of cancer^25^. A median time to development of PE after lung cancer diagnosis of 3.5 months (1–6.5 months) has previously been reported^22^. Majority of patients in the current study (86.8%) developed PE within 3 months after the diagnosis of lung cancer, which might further increase the rate of mortality. In addition, in our study, 69.8% of patients with lung cancer were in stages III and IV when PE was diagnosed, which decreased the survival time further. In the CANTARISK study, high risk of VTE (VTE-RS score ≥3) was associated with a markedly increased risk of early mortality (*HR* = 2.076, 95%*CI*: 1.607–2.681)^33^. Furthermore, patients with lung cancer and PE have been shown to have a significantly shorter survival time compared with patients without PE (243.5 d *vs*. 327 d, *P* = 0.01)^25^. Similarly, the median survival time of PE patients has been reported to be shorter than that of non-PE patients (6.65 months *vs*. 17.0 months)^22^. Furthermore, compared with patients with asymptomatic PE, those with symptomatic PE have been shown to have a markedly shorter median survival time (2.8 months *vs*. 7.2 months)^34^. Consistent with previous studies, we found that older patients with lung cancer and PE had significantly shorter median survival time compared with those without PE (4.3 months *vs*. 9.2 months). However, the median tumor-related survival time was similar between the two groups (7.1 months *vs*. 9.7 months, *P* = 0.165). The higher mortality associated with PE indicates the necessity to identify accurate predictors of PE to lower the incidence in high-risk patients with lung cancer^35^.

Patients in this study had multiple risk factors for PE. Previous studies have identified that advanced age is a risk factor for PE^36^. The results of our study were consistent with previous findings, with advanced age (≥77 years) found to be associated with a 2.58-fold increased risk for PE. However, in a previous study conducted in Japan among 235,104 patients with lung cancer treated with platinum-based chemotherapy, 675 had VTE during hospitalization. Patients older than 70 years were at the lowest risk for VTE (*OR* = 0.59, 95%*CI*: 0.44–0.79), which might be explained by differences in dose and duration of chemotherapy between older and younger patients^21^. Several studies have confirmed that patients with PE have a significantly higher D-dimer level, and PE could potentially be ruled out in patients with a negative D-dimer result (<500 mg/L) and a Well score of 4 or less^37^. However, older patients presenting with PE had an increased level of D-dimer, and the age-adjusted D-dimer, which is calculated as the patient age multiplied by 10 mg/L in patients older than 50 years^38^, is associated with a 5% absolute increased efficiency compared with a fixed threshold (500 mg/L)^39,40^. Additionally, advanced stage of lung cancer has been shown to be related to higher risk of PE^41^. We also found that clinical stage III–IV was an independent risk factor for PE in older patients (OR = 2.21, 95%*CI*: 1.03–4.74). Additionally, several studies have demonstrated that patients with adenocarcinoma of the lung had a significantly higher risk of venous thromboembolic events^42,43^. Consistent with previous studies, we found that the most common histologic type in our patient population was adenocarcinoma (52.8%), which was also an independent risk factor for PE. Furthermore, hypoxemia can increase the risk of VTE events^43^. Among patients with lung cancer, PaO_2_ was previously identified as a risk factor for PE (OR = 2.7, 95%CI: 1.31–3.98)^22^, which was consistent with our findings. Therefore, advanced age, clinical stage III–IV, adenocarcinoma, high D-dimer level, and low PaO_2_ were identified as independent risk factors for PE in the present analysis.

Treatment of VTE in patients with cancer is challenging. Balancing the risk of bleeding to the thrombosis is complicated by a variety of factors. In the landmark CLOT trial including 672 patients with cancer and acute symptomatic VTE, Lee *et al*. found that dalteparin was associated with significantly decreased rate of recurrent VTE and similar risk of major bleeding compared with oral-anticoagulant^44^. American College of Chest Physicians Evidence-Based Clinical Practice (ACCP) and European Society of Medical Oncology (ESMO) guidelines recommend low molecular weight heparin (LMWH) as the preferred anticoagulant in VTE prevention and treatment for patients with cancer^45,46^. The direct oral anticoagulants (NOACs) such as dabigatran, rivaroxaban, edoxaban, apixaban have been approved for VTE treatment in the general population^47–49^. Recently, several studies demonstrated that NOACs were an alternative anticoagulant for the prevention and treatment of VTE in patients with cancer. The RECOVER and RECOVER II trials showed that the rate of recurrent VTE or VTE-related mortality was not significant different between dabigatran and warfarin groups in patients with cancer (3.5% vs 4.7%)^47^. The EINSTEIN-DVT and EINSTEIN-PE trials demonstrated also showed similar VTE recurrence among patients treated with rivaroxaban and those treated with a LMWH and Vitamin-K antagonist^50^. In the AMPLIFY trial, recurrent VTE was comparable among patients with cancer treated with apixaban and those treated with enoxaparin/warfarin (3.7% vs 6.4%)^48^. In the HOKUSAI-VTE trial, oral edoxaban was associated with similar rate of VTE recurrence and major bleeding compared with dalteparin in patients with cancer-associated VTE^51^. Therefore, the clinical effect of NOACs for VTE prevention and treatment is still uncertain. Many ongoing trials such as CAP trial, CARAVAGGIO trial, CASTA-DIVA trial, CANVAS trial is evaluating the safety and efficacy of NOACs in cancer patients.

### Limitations

This study has several limitations. First, the use of PSM method for patient selection is a potential weakness. There might be remaining imbalances in terms of confounding^52^. Second, ours was a relatively small cohort study, meaning that the primary analysis may have been underpowered and these finding require further confirmation. Third, this was a single-center study. Therefore, selection bias might have occurred during identification of the patients, and our data cannot be directly extrapolated to other health centers. Fourth, the REVERSE study showed that the residual PE after 5–7 months of oral anticoagulant therapy was a predictor for recurrence^53^. However, we did not evaluate the effect of residual PE after the anticoagulant and/or thrombolysis therapy on the primary and secondary outcomes. Fifthly, the study analyzed the characteristics of cancer associated PE in the past 10 years, and we cannot exclude that accuracy of CTPA, venous ultrasound and anticoagulant and anti-tumor treatment options used in our study may influence our results. Finally, most patients were treated with LMWH or warfarin, we cannot comment on whether NOACs would affect the results^54^. Therefore, due to the limitations of this study discussed above, further studies are required to verify the present findings.

## Conclusion

In conclusion, older patients with lung cancer complicated with PE have atypical clinical manifestations and high mortality. Advanced age, clinical stage III–IV, adenocarcinoma, high serum D-dimer, and low PaO_2_ are independent risk factors for PE. Early prevention and treatment of PE in high-risk populations is needed to improve the prognosis of these patients.

## References

[1] Falanga A, Russo L, Milesi V, Vignoli A (2017). Mechanisms and risk factors of thrombosis in cancer. Crit Rev Oncol Hematol.

[2] Baron JA, Gridley G, Weiderpass E, Nyren O, Linet M (1998). Venous thromboembolism and cancer. Lancet.

[3] Prandoni P, Falanga A, Piccioli A (2005). Cancer and venous thromboembolism. Lancet Oncol.

[4] Christensen TD (2014). Venous thromboembolism in patients undergoing operations for lung cancer: a systematic review. Ann Thorac Surg.

[5] Browne AM (2010). Unsuspected pulmonary emboli in oncology patients undergoing routine computed tomography imaging. J Thorac Oncol.

[6] Khorana AA, Francis CW, Culakova E, Kuderer NM, Lyman GH (2007). Thromboembolism is a leading cause of death in cancer patients receiving outpatient chemotherapy. J Thromb Haemost.

[7] Wang H, Xu X, Pu C, Li L (2019). Clinical characteristics and prognosis of cancer patients with venous thromboembolism. J Cancer Res Ther.

[8] Blom JW, Doggen CJ, Osanto S, Rosendaal FR (2005). Malignancies, prothrombotic mutations, and the risk of venous thrombosis. JAMA.

[9] Shinagare AB (2011). Incidence of pulmonary embolism in oncologic outpatients at a tertiary cancer center. Cancer.

[10] Cao M, Chen W (2019). Epidemiology of lung cancer in China. Thorac Cancer.

[11] Chen W (2016). Cancer statistics in China, 2015. CA Cancer J Clin.

[12] Garcia Gomez LC (2019). Pulmonary embolism in very elderly patients. A diagnostic challenge. Rev Clin Esp.

[13] Kiluk IE (2017). Different manifestations of pulmonary embolism in younger compared to older patients: Clinical presentation, prediction rules and long-term outcomes. Adv Med Sci.

[14] Tarbox AK, Swaroop M (2013). Pulmonary embolism. Int J Crit Illn Inj Sci.

[15] Lange N (2019). Anticoagulation quality and clinical outcomes in multimorbid elderly patients with acute venous thromboembolism. Thromb Res.

[16] Haddad TC, Greeno EW (2006). Chemotherapy-induced thrombosis. Thromb Res.

[17] Austin PC (2011). Optimal caliper widths for propensity-score matching when estimating differences in means and differences in proportions in observational studies. Pharm Stat.

[18] Tsai AW (2002). Cardiovascular risk factors and venous thromboembolism incidence: the longitudinal investigation of thromboembolism etiology. Arch Intern Med.

[19] Singh G, Rathi AK, Singh K, Sharma D (2017). Venous thromboembolism in cancer patients - magnitude of problem, approach, and management. Indian J Cancer.

[20] Guy JB (2017). Venous thromboembolism in radiation therapy cancer patients: Findings from the RIETE registry. Crit Rev Oncol Hematol.

[21] Mitani A (2018). Venous thromboembolic events in patients with lung cancer treated with cisplatin-based versus carboplatin/nedaplatin-based chemotherapy. Anticancer Drugs.

[22] Li M, Guo Q, Hu W (2019). Incidence, risk factors, and outcomes of venous thromboembolism after oncologic surgery: A systematic review and meta-analysis. Thromb Res.

[23] Kang JR (2017). Peripherally Inserted Central Catheter-Related Vein Thrombosis in Patients With Lung Cancer. Clin Appl Thromb Hemost.

[24] Essien EO, Rali P, Mathai SC (2019). Pulmonary Embolism. Med Clin North Am.

[25] Chuang YM, Yu CJ (2009). Clinical characteristics and outcomes of lung cancer with pulmonary embolism. Oncology.

[26] Li G, Li Y, Ma S (2017). Lung Cancer Complicated With Asymptomatic Pulmonary Embolism: Clinical Analysis of 84 Patients. Technol Cancer Res Treat.

[27] John M, Greenwald DT, Nicholson BL, Kemper SE (2014). Long-term outcomes in individuals aged 75 and older with pulmonary embolism. J Am Geriatr Soc.

[28] Moutzouris JP (2014). Acute pulmonary embolism in individuals aged 80 and older. J Am Geriatr Soc.

[29] Doherty S (2017). Pulmonary embolism An update. Aust Fam Physician.

[30] Olie V (2015). Time trends in pulmonary embolism mortality in France, 2000–2010. Thromb Res.

[31] Goldhaber SZ, Bounameaux H (2012). Pulmonary embolism and deep vein thrombosis. Lancet.

[32] Dentali F (2008). Incidence of venous thromboembolism in patients undergoing thoracotomy for lung cancer. J Thorac Cardiovasc Surg.

[33] Kuderer NM (2018). Predictors of Venous Thromboembolism and Early Mortality in Lung Cancer: Results from a Global Prospective Study (CANTARISK). Oncologist.

[34] Li YP (2018). Prevalence and Risk Factors of Acute Pulmonary Embolism in Patients with Lung Cancer Surgery. Semin Thromb Hemost.

[35] Jimenez D, Lobo JL, Barrios D, Prandoni P, Yusen RD (2016). Risk stratification of patients with acute symptomatic pulmonary embolism. Intern Emerg Med.

[36] Minges KE (2015). National Trends in Pulmonary Embolism Hospitalization Rates and Outcomes for Adults Aged>/=65 Years in the United States (1999 to 2010). Am J Cardiol.

[37] van Belle A (2006). Effectiveness of managing suspected pulmonary embolism using an algorithm combining clinical probability, D-dimer testing, and computed tomography. JAMA.

[38] Righini M (2014). Age-adjusted D-dimer cutoff levels to rule out pulmonary embolism: the ADJUST-PE study. JAMA.

[39] Schouten HJ (2013). Diagnostic accuracy of conventional or age adjusted D-dimer cut-off values in older patients with suspected venous thromboembolism: systematic review and meta-analysis. BMJ.

[40] van Es N (2017). The original and simplified Wells rules and age-adjusted D-dimer testing to rule out pulmonary embolism: an individual patient data meta-analysis. J Thromb Haemost.

[41] Li R (2016). Advanced nodal stage predicts venous thromboembolism in patients with locally advanced non-small cell lung cancer. Lung Cancer.

[42] Tesselaar ME, Osanto S (2007). Risk of venous thromboembolism in lung cancer. Curr Opin Pulm Med.

[43] Huet Y (1985). Hypoxemia in acute pulmonary embolism. Chest.

[44] Lee AY (2003). Low-molecular-weight heparin versus a coumarin for the prevention of recurrent venous thromboembolism in patients with cancer. N Engl J Med.

[45] Holbrook A (2012). Evidence-based management of anticoagulant therapy: Antithrombotic Therapy and Prevention of Thrombosis, 9th ed: American College of Chest Physicians Evidence-Based Clinical Practice Guidelines. Chest.

[46] Mandala M, Falanga A, Roila F, Group EGW (2011). Management of venous thromboembolism (VTE) in cancer patients: ESMO Clinical Practice Guidelines. Ann Oncol.

[47] Schulman S (2009). Dabigatran versus warfarin in the treatment of acute venous thromboembolism. N Engl J Med.

[48] Agnelli G (2013). Apixaban for extended treatment of venous thromboembolism. N Engl J Med.

[49] Weitz JI (2017). Rivaroxaban or Aspirin for Extended Treatment of Venous Thromboembolism. N Engl J Med.

[50] Prins MH (2014). Oral rivaroxaban versus enoxaparin with vitamin K antagonist for the treatment of symptomatic venous thromboembolism in patients with cancer (EINSTEIN-DVT and EINSTEIN-PE): a pooled subgroup analysis of two randomised controlled trials. Lancet Haematol.

[51] Raskob GE (2018). Edoxaban for the Treatment of Cancer-Associated Venous Thromboembolism. N Engl J Med.

[52] Staffa SJ, Zurakowski D (2018). Five Steps to Successfully Implement and Evaluate Propensity Score Matching in Clinical Research Studies. Anesth Analg.

[53] Wan T (2018). Residual pulmonary embolism as a predictor for recurrence after a first unprovoked episode: Results from the REVERSE cohort study. Thromb Res.

[54] Dentali F (2015). Non-vitamin K oral anticoagulants in patients with pulmonary embolism: a systematic review and meta-analysis of the literature. Intern Emerg Med.

